# Association between GPx‐1 polymorphisms and personality traits in healthy Chinese‐Han subjects

**DOI:** 10.1002/brb3.1897

**Published:** 2020-10-17

**Authors:** Xiaojun Shao, Dongxue Sun, Bihui Zhang, Lingfei Cheng, Ci Yan, Gang Zhu

**Affiliations:** ^1^ Department of Psychiatry The First Affiliated Hospital of China Medical University Shenyang China; ^2^ Central Laboratory The First Affiliated Hospital of China Medical University Shenyang China

**Keywords:** Chinese, gene polymorphism, glutathione peroxidase 1, personality trait

## Abstract

**Objectives:**

Cloninger developed the three‐dimensional personality theory and Tridimensional Personality Questionnaire (TPQ), which shows that some dimensions of personality traits are heritable and related to neurotransmitters including dopamine. glutathione peroxidase 1 (GPx1) plays an important role in metabolic dopamine change and closely relates to neurological and psychiatric disorders. The impact of GPx‐1 polymorphisms has been rarely explored in the field of personality traits. We decide to explore the relationships between them in healthy Chinese‐Han subjects by using the polymerase chain reaction‐restriction fragment length polymorphism (PCR‐RFLP).

**Methods:**

In our study, 493 healthy Chinese‐Han participants (male = 234, female = 259) were recruited. 2 ml of EDTA‐treated blood from each volunteer was taken; meanwhile, personality traits were assessed by TPQ. We detected the genotypes of selected two polymorphisms through PCR‐RFLP after extracting DNA. Finally, the association between different genotypes and TPQ scores was performed using SPSS, *p* < .05 is seen as significant statistical significance.

**Results:**

Our data found a correlation between rs1800668 and novelty seeking (NS) subscale NS2 (*X* ^2^ = 7.392, *p* = .025). While the results showed the rs1050450 was significantly associated with NS4 (*X* ^2^ = 6.059, *p* = .048). Regarding sex stratification, there was a significant difference in the NS2 score (*X* ^2^ = 8.232, *p* = .016) among women for rs1800668. No sex effect was observed for either genotype for rs1050450.

**Conclusion:**

GPx‐1polymorphism is related to personality traits in healthy Chinese‐Han subjects. Our results suggested that GPx‐1 may be involved in the biological mechanisms and be a potential gene that influenced personality.

## Graphical abstract

This study is the first to thoroughly investigate associations between GPx‐1 polymorphisms and personality traits. Our results demonstrated that rs1050450 was significantly associated with the disorderliness trait. The rs1800668 polymorphism in GPx‐1 affected the personality trait of impulsiveness in healthy Chinese‐Han subjects.

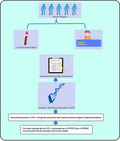

## 1 INTRODUCTION

Personality traits determine the basic behaviors of the individual, which are the relatively stable and persistent tendencies of the individual to act in certain ways (Roberts, 2009). These traits, the core component of personality, can reduce personal volatility and lead to undertaking specific and effective actions. Based on the theory of biological social personality, Cloninger developed the three‐dimensional personality theory and its questionnaire (Tridimensional Personality Questionnaire; TPQ), which shows that some dimensions of personality are heritable and related to neurobiological markers (Cloninger, 1987). In fact, psychologists in the field of personality research have reached a consensus that personality is formed by interactions between heredity and environment (Roberts, 2018; Soliemanifar et al., 2018). Cloninger divided personality into three dimensions, namely novelty seeking (NS), harm avoidance (HA), and reward dependence (RD). The three dimensions are associated with three neurotransmitters, dopamine (DA), serotonin (5‐HT), and norepinephrine (NA) (Cloninger, 1987, 1988). Dellu et al. confirmed that individual differences in the function of dopamine might be the basis for humans to show the personality trait of NS (Dellu et al., 1996). Subsequent work reported in the midbrain low D2/3 receptor (D2/3–R) availability, which is associated with elevated DA release in the striatum, could predict impulsivity by using PET (Buckholtz et al., 2010). In rats, the low novelty trend is related to low extracellular DA levels of striatum, agreeing with human data (Mallo et al., 2007). Recently, a review of the relationship between temperamental traits and monoamine neurotransmitters indicated that DA contributes to the complex involvement of impulsivity, NS, and incentive motivation (Robbins, 2018). A similar conclusion from PET studies (Farde et al., 2018) illustrated possible associations between DA with NS and impulsivity; however, these results may be related to psychoticism and social withdrawal. Additionally, previous studies have shown that the dopamine receptor D4 (DRD4) is related to personality traits (Ebstein et al., 1996). Recent studies in our laboratory have demonstrated that personality traits are associated with Neurotensin receptor 1 (NTR1) (Ma et al., 2014) and DARPP‐32 (Li et al., 2011), which can affect the dopaminergic system. Taken together, these studies strongly confirmed that personality traits are related to monoamine neurotransmitters, especially DA.

In organisms, glutathione peroxidase (GPx) is a key reactive oxygen species (ROS) scavenger (Do et al., 2019) that combines with catalase (CAT) and superoxide dismutase (SOD) to form an antioxidant defense system (Tolomeo et al., 2016). GPx likes a reducing agent to eliminate hydrogen peroxide (H_2_O_2_) and lipid peroxides, preventing peroxidative damage to the cell membrane and other organelles by using glutathione (GSH) (Hansen et al., 2006; Sies, 1999). GPx is responsible for recycling GSH (Salminen & Paul, 2014) as well as regulating GSH regeneration (Chance et al., 1979). GSH is an electron donor in the redox process of GPx (Dringen, 2000). GPx‐1 is one of the five GPx homologues is the most widely distributed enzyme in the family, and is especially abundant in vascular endothelial cells (Forgione et al., 2002). The GPx‐1 locus is located on chromosome 3p21.3 (Kiss et al., 1997) and contains two exons and one intron (Ishida et al., 1987). In cells exposed to ROS, GPx‐1 showed superior protection against SOD and CAT (Takeshita et al., 2000).

There is considerable evidence that GPx has a strong association with some diseases, many with links to the DA system (Bai et al., 2017; Cardoso et al., 2012; Gardaneh et al., 2011; Paz‐y‐Mino et al., 2010; Power & Blumbergs, 2009). In fact, DA is a major source of ROS in the central nervous system, as it can produce ROS and DA‐quinone during its autooxidation (Zucca et al., 2014). GPx is expressed in neurons and glial cells and is involved in the endogenous response to central nervous system oxidative stress as a key enzyme in the antioxidant system (Salminen & Paul, 2014) in the brain; GPx is also closely related to neurological and psychiatric disorders. Studies have found that in Alzheimer's disease (AD), degeneration of the central cholinergic system and free radical metabolism are gradually aggravated, and the activities of SOD and GPx in hippocampus, which are closely related to the system, are significantly decreased over time (Pocernich & Butterfield, 2012). It has also been reported in animal experiments that the activities of GPx and reductase in AD‐related APP/PS‐1 mice treated with antioxidants before induced oxidation were higher than those in controls. This treatment could significantly reduce oxidative stress and improve the cognitive impairment AD model mice (Huang et al., 2010). Vincent et al. reported that low GSH and ascorbic acid levels in the brain are associated with dopaminergic system disorders, which are implicated in the development and progression of cognitive impairment in schizophrenia (Castagné et al., 2004). Buckman et al. found that brain atrophy measured after head CT scan in patients with chronic schizophrenia was negatively correlated with GSH‐px activity in the platelets, which was more significant in patients with schizophrenia with negative symptoms as the core clinical manifestation (Buckman et al., 1990). Studies have shown that in Parkinson's disease (PD) patients, GSH loss occurs in the cell bodies and dendritic processes of dopaminergic nigral neurons (Pearce et al., 1997), and total PD GSH is selectively exhausted in the early stages of PD (Bannon et al., 1984; Sian et al., 1994).

Currently, a tremendous amount of domestic and foreign scientific research have proven that GPx‐1 polymorphisms are associated with different diseases, such as coronary artery disease (Kiss et al., 1997; Yeh et al., 2018), cancer (Habyarimana et al., 2018; Lubos et al., 2011), diabetes and its vascular complications (Buraczynska et al., 2017; Sultan et al., 2018), rheumatoid arthritis (Irfan et al., 2016), Huntington's disease (HD) (Zheng et al., 2018), PD (Gardaneh et al., 2011; Redensek et al., 2019), autism (Ming et al., 2010), and postpartum hemorrhage (Endler et al., 2016). However, research into the involvement of GPx‐1 in human personality traits is lacking.

The types of GPx‐1 SNPs in different populations are as follows: Prol98Leu (rs1050450) in Exon 2, −602A/G and 2T/C in the promoter region, and Ala5/Ala6/Ala7 repeat polymorphisms in Exon 1 (Hamanishi et al., 2004). Additionally, there are 592G/A (Ratnasinghe et al., 2000), −641A/G (rs3811699), and −46C/T (rs1800668) (Lubos et al., 2011) polymorphisms, among others. Studies have shown that GPx‐1 polymorphisms, especially rs1800668, can affect the activity of the GPx‐1 promoter and that the C allele is related to increased GPx activity (Lubos et al., 2011; Najafi et al., 2012), which may play a role in improving antioxidant activity. The variation at nucleotide 593 (rs1050450) creates a C ≥ T allele in Exon 2, leading to a proline to leucine (Ratnasinghe et al., 2000) substitution at codon 198, which leads to decreased GPx‐1 activity (Hong et al., 2013). This study detected GPx‐1 single‐nucleotide polymorphisms (SNPs) (rs1800668 and rs1050450), which were selected based on a vast multitude of literature, by the polymerase chain reaction‐restriction fragment length polymorphism (PCR‐RFLP) and explored whether there was a correlation between GPx‐1 polymorphisms and personality traits in a healthy Chinese‐Han population.

## 2 METHODS

This study was approved by the Ethics Committee of China Medical University and was based on the Declaration of Helsinki as the moral principle. Informed consent was obtained from all individual participants included in the study.

Healthy Chinese‐Han subjects without any mental disorders, neurological or physical diseases were recruited as volunteers. All subjects were recruited from medical staff and students of our hospital. All subjects underwent a complete interview to exclude psychiatric disorders. The rs1800668 sample was composed of 493 subjects (male = 234, female = 259) with an average age of 30.54 ± 7.85 years. Meanwhile, the rs1050450 group comprised 463 subjects (male = 223, female = 240), with a mean age of 30.03 ± 7.83 years. For genomic DNA isolation, 2 ml of EDTA‐treated blood from each volunteer was taken for DNA isolation using a DNA extraction kit (Wizard Genomic DNA purification kit, Thermo Fisher Scientific). Detection of two single‐nucleotide polymorphisms, including rs1800668 and rs1050450, was performed by a PCR‐based amplification strategy. RFLP analysis was carried out using the restriction enzymes HpyCH4III and BlpI (New England Biolabs, Ipswich, MA, USA). The Chinese version of TPQ, a self‐rating scale that consists of 100 "yes" or "no" items, was also given to all volunteers. All subjects were asked to complete the highly reliable Chinese version questionnaire by themselves within 30 min, which was used for measuring the 3 temperament dimensions of personality traits (NS, HA, and RD).

PCR primers were assembled by a local representative of Takara Biotechnology. The forward primer 5′‐CAGCCTCCTATGCCAAACC‐3' and reverse primer 5'‐GCTCGTTCATCTGGGTGTAGTC‐3' were used to amplify rs1800668; the forward primer 5'‐CTCCCTTGTTTGTGGTTAG‐3' and reverse primer 5'‐CCTCCCTCGTAGGTTTAG‐3' were used to amplify rs1050450. The annealing temperatures of the primers were 62.4°C (rs1800668) and 55°C (rs1050450). The sizes of PCR products were 561 bp (rs1800668) and 529 bp (rs1050450). A 2% agarose gel was used to resolve PCR products and digested products, which were visualized using a UV Transilluminator. The lengths of restriction digest products were 330/231 bp (rs1800668 C/T) and 419/110 bp (rs1050450 C/T). For rs1800668, CC and TT homozygotes showed two fragments (330 and 231 bp) and a fragment (561 bp), and CT showed three fragments 561, 330, and 231 bp. For rs1050450, two fragments of 419 and 110 bp for CC homozygous, three fragments of 529, 419, and 110 bp for CT heterozygote, while the uncleaved PCR product 529 bp for TT homozygous.

The chi‐square test was used to analyze Hardy–Weinberg equilibrium (HWE), gene frequencies, and allele frequencies. The Kruskal–Walis test was used to analyze the relationship between SNP genotypes and TPQ scores. All tests performed using SPSS v17.0 for windows (SPSS Inc.).

## 3 RESULTS

Table 1 describes the results for HWE estimation along with genotype and allele frequencies. These data indicated that the two selected SNPs (rs1800668 and rs1050450) were in accordance with the genetic balance of HWE; thus, the selected samples were representative of the population.

**TABLE 1:** Genotype and allele frequencies of GPx‐1 rs1800668 and rs1050450

SNP	Allele	*N* (%)	χ^2^	*p* value
rs1800668	TT	5 (1.0%)	3.469	.063
	CT	58 (11.8%)		
	CC	430 (87.2%)		
rs1050450	TT	5 (1.1%)	1.845	.174
	CT	63 (13.6%)		
	CC	395 (85.3%)		

Note

*p* < .05 (indicates statistical significance).

Tables 2 and 3 describe the effects of GPx‐1 polymorphisms on TPQ scores by median and interquartile range. There was no significant difference in the scores of rs1800668, NS (*X* ^2^ = 0.09, *p* = .956), HA (*X* ^2^ = 0.715, *p* = .999), RD (*X* ^2^ = 0.543, *p* = .762), but there was a difference in the scores for NS2 (*X* ^2^ = 7.392, *p* = .025). The CT genotype was found to have significantly higher NS2 scores than the other two genotypes. Table 3 indicated that significant associations existed between the rs1050450 polymorphism and personality traits regarding NS4 scores (*X* ^2^ = 6.059, *p* = .048), while the TT genotype had higher scores than the CC/CT genotype.

**TABLE 2:** Effects of GPx‐1 rs1800668 polymorphism on TPQ scores

Genotype *n* (%)	TT 5（1%）	CC 430（87.2%）	CT 58（11.8%）	*χ* ^2^	*p*
NS	15(9.5–17)	14 (12–17)	14.5 (11.75–17)	0.09	.956
NS1	4 (2.5–5)	4 (3–5)	4 (2–6)	0.309	.857
NS2	2 (0–3)	3 (2–5)	4 (3–5)	7.392	.025*
NS3	5 (3–5)	4 (3–5)	4 (3–5)	0.815	.665
NS4	4 (2.5–5.5)	3 (2–5)	3 (2–4)	3.927	.14
HA	12 (10.5–16)	14 (11–17)	14(11.75–16.25)	0.715	.699
HA1	4 (2–6)	4 (3–5)	4 (2–5)	0.6	.741
HA2	4 (3–4.5)	4 (3–5)	3 (3–4)	3.405	.182
HA3	2 (0.5–2.5)	2 (1–3)	2 (1–4)	1.785	.41
HA4	4 (3–4)	4 (3–5)	4 (2–5)	0.712	.701
RD	19 (16–19)	19 (17–21)	18.5 (17–20.25)	0.543	.762
RD1	4 (3.5–5)	4 (4–5)	5 (4–5)	1.003	.606
RD2	4 (3–5.5)	5 (3–6)	4 (3–5.25)	0.978	.613
RD3	7 (5–8.5)	7 (6–8)	7 (6–8.25)	0.035	.982
RD4	2 (1.5–4)	3 (2–4)	3 (2–4)	0.866	.649

* *p* < .05 (indicates statistical significance).

**TABLE 3:** Effects of GPx‐1 rs1050450 polymorphism and TPQ scores

Genotype	CC	TT	CT	*χ* ^2^	*p*
*n* (%)	395 (85.3%）	5 (1.1%）	63 (13.6%）		
NS	14 (12–17)	15 (9.5–19)	14 (11–17)	0.734	.693
NS1	4 (2–5)	4 (2.5–4.5)	4 (2–6)	0.265	.876
NS2	3 (2–5)	2 (0–4)	4 (3–5)	4.517	.105
NS3	4 (3–5)	5 (3.5–5.5)	3 (3–4)	2.814	.245
NS4	3 (2–5)	5 (2.5–5.5)	3 (2–4)	6.059	.048*
HA	14 (11–17)	12 (10.5–17.5)	14 (12–16)	0.681	.712
HA1	4 (3–5)	5 (2–6)	3 (2–5)	1.381	.501
HA2	4 (3–5)	4 (3–5.5)	3 (3–4)	3.706	.157
HA3	2 (1–3)	1 (0.5–2.5)	2 (1–4)	1.502	.472
HA4	4 (3–5)	4 (3–4.5)	4 (2–5)	0.215	.898
RD	19 (17–21)	19 (17–20)	19 (17–21)	0.017	.992
RD1	4 (4–5)	5 (3.5–5)	5 (4–5)	1.982	.371
RD2	4 (3–6)	4 (3.5–5.5)	4 (3–6)	0.156	.925
RD3	7 (6–9)	7 (5–8.5)	7 (6–8)	0.083	.959
RD4	3 (2–4)	4 (1.5–4)	3 (2–4)	0.416	.812

* *p* < .0.5 (indicates statistical significance).

When analyzed separately by sex, we found no TT genotype in males for rs1800668, so we only analyzed the CT and CC genotypes. There was no significant difference in TPQ scores among the different genotypic groups in males. However, the RD1 score (*X* ^2^ = 3.85, *p* = .05) suggested that there might be genotypic differences (no other data given). Table 4 shows that there was a significant difference in NS2 scores among women (*X* ^2^ = 8.232, *p* = .016) describing by median and interquartile range. At the same time, females with the combined genotype CT for rs1800668 had higher NS2 scores than the CC and TT genotypes. No sex effect was observed for either rs1050450 genotype (data not shown).

**TABLE 4:** Effects of GPx‐1 rs1800668 polymorphism on TPQ scores in females

Genotype	TT	CC	CT	*X* ^2^	*p*
*n* (%)	5 (1.9%)	220 (84.9%)	34 (13.1%)		
NS	15 (9.5–17)	14.5 (12–18)	15.5(11–19.25)	0.358	.836
NS1	4 (2.5–5)	4 (3–6)	4.5 (2–6)	0.234	.89
NS2	2 (0–3)	3 (2–5)	4 (3–5)	8.232	.016*
NS3	5 (3–5)	4 (3–5)	4 (3–5)	1.517	.468
NS4	4 (2.5–5.5)	3 (2–5)	3 (2–4)	3.002	.223
HA	12 (10.5–16)	14 (11–18)	13 (11–18)	0.779	.677
HA1	4 (2–6)	4 (2.25–5)	3 (1.75–5)	2.369	.306
HA2	4 (3–5.5)	4 (3–5)	3.5 (3–4)	1.999	.368
HA3	2 (0.5–2.5)	2 (1–3)	2 (1–4)	1.276	.528
HA4	4 (3–4)	4 (3–5)	4 (2–5.25)	1.198	.549
RD	19 (16–19)	19 (17–21)	19 (17–21)	1.338	.512
RD1	4 (3.5–5)	4 (4–5)	4 (4–5)	0.138	.933
RD2	4 (3–5.5)	5 (3–6)	4.5 (3–6)	0.447	.8
RD3	7 (5–8.5)	7 (6–9)	7 (6–9)	0.144	.931
RD4	2 (1.5–4)	3 (2–4)	3.5 (2–4)	1.207	.547

* *p* < .05 (indicates statistical significance).

## 4 DISCUSSION

In this study, rs1050450 and rs1800668 were selected to evaluate associations between selected SNPs and personality traits in healthy Chinese‐Han subjects as well as the effect of sex. The principal findings of this study were that the GPx‐1 polymorphisms rs1050450 and rs1800668 were significantly associated with NS in a healthy Chinese‐Han population. These results are consistent with several previous studies on the relationship between GPx‐1 and DA, and the relationship between the NS scale and DA in TPQ.

The NS trait consists of impulsive responses and exploratory behaviors, which are accompanied by a strong exciting and novel emotional experience when looking for novel and beneficial stimuli. Individuals with high NS scores are excitable, impulsive, and grumpy. NS showed a strong genetic tendency (Garcia et al., 2013); it has been estimated that the heritability of NS behavior is 41% (Heath et al., 1994). In Cloninger's theory, NS is a multifaceted essential feature composed of four aspects. This dimension is composed of exploratory excitability versus stoic rigidity (NS1), impulsiveness versus reflection (NS2), extravagance versus reserve (NS3), and disorderliness versus regimentation (NS4). Among them, NS1 and NS2 reflect the uniqueness of the NS dimension. Cloninger proposed that the NS dimension is associated with DA, such as the DRD4 gene mentioned earlier. Several studies (Caravaggio et al., 2016; Gjedde et al., 2010) that examined striatum DA in healthy individuals also found that the neural dopamine D2/3 receptor (D2/3R) was associated with personality traits of impulsivity in the pursuit of NS by using positron emission tomography. Lawrence et al. reported a significant correlation between DA synthesis ability in the ventral striatum and NS3 (Extravagance vs. Reserve) (Lawrence & Brooks, 2014). Joyce et al. confirmed that the 9‐repeat allele of the DA transporter was associated with the personality characteristics of impulsive anger, and the basis of a dopaminergic neurobiological model indicated that this correlation might form the personality traits of impulsive anger (Joyce et al., 2009). Current studies have indicated that DA was a major regulator of human NS behavior.

Among the existing reports of GPx‐1 SNPs, rs1050450 has been the most extensively studied. A study from Turkey showed that this polymorphism is not associated with risk of panic disorder; however, the sex‐specific role of the GPx‐Pro198Leu allele may be related to PD development (Cengiz et al., 2015). A study of the Ecuadorian population (Paz‐y‐Mino et al., 2010) showed that the Gx1‐Leu allele increased the risk of AD. Interestingly, Cardoso et al. did not observe a difference in the genotypic frequency between AD patients and the control groups in a Brazilian population (Cardoso et al., 2012). However, a case–controlled study on the association of GPx‐1 and GPx4 polymorphisms with episodic memory and AD in southern Brazil found that TT homozygotes showed a lower score for long‐term visual memory, but could be related to lower risk for dementia (da Rocha et al., 2018). Another study measured GPx‐1 activity (with respect to rs1050450) in human erythrocyte extracts and reported lower activity in TT extract than CT/CC extract (Ravn‐Haren et al., 2006). And in this study, we found that TT homozygotes got the highest score in the NS subscale NS4 (disorderliness vs. regimentation).

Daisuke Matsuzawa et al. determined an association between the rs1050450 polymorphism and the personality trait of openness to experience on the Revised NEO Personality Inventory in healthy Japanese individuals and observed that the CC genotype presented significantly higher scores than those in CT genotype and TT genotype. Furthermore, no statistical difference among the three groups on the Temperament and Character Inventory, which is a 240‐item self‐report instrument that measures four dimensions of temperament (novelty seeking, harm avoidance, reward dependence, and persistence) and three dimensions of character (self‐directedness, cooperativeness, and self‐transcendence) (Matsuzawa et al., 2005). In contrast, we observed that the rs1050450 polymorphism was significantly associated with NS4. One of the reasons for these results might be the racial and ethnic difference between the study populations. Another crucial point to consider is the limited sample size who were examined in the study mentioned above. Our study consisted of 223 men and 240 women, a total of 463 individuals, almost three times the sample size of the previous study. Further research is needed to explore whether there are associations between GPx‐1 polymorphisms and personality traits among different ethnic population. Taken together, our study points out that we are the first to show that rs1050450 polymorphism was significantly related to the personality trait of disorderliness in NS, which may be due to the fact that rs1050450 can reduce GPx‐1 activity, and thus affect DA signaling.

To the best of our knowledge, no studies have investigated the correlation between the GPx‐1 rs1800668 polymorphism and personality traits at this stage, and only a few of studies have analyzed the association between rs1800668 and certain diseases. A study conducted in a Chinese‐Han population showed that individuals with the rsl800668 CC and CT genotypes were at high risk for noise‐induced hearing loss and had a higher risk of carrying the CT allele (Wen et al., 2014). Irfan et al. observed that the CT genotype of GPx‐1 increased the risk of rheumatoid arthritis. The TT genotype is involved in the pathogenesis of rheumatoid arthritis, while the CC genotype had protective effect (Irfan et al., 2016). No significantly high‐risk association was reported with sensitivity‐related illnesses and GPx1 rs1800668 polymorphism; meanwhile, significant differences were found in healthy subjects with different genotypes, since genotype TT had the highest levels of oxidative stress (Gugliandolo et al., 2016).

In this study, we found that for rsl800668 there were differences in TPQ NS2 scores for each genotype (*X* ^2^ = 7.392, *p* = .025), with the highest being the CT genotype score and the lowest being the TT genotype score. The TT genotype was not observed in male subjects after sex stratification. The scores of male RD1 (*X* ^2^ = 3.85, *p* = .05) suggested that there might be differences among the genotypes, among which the CT genotype showed the highest score. The results of this study need to be further verified in a larger sample size to continue to explore the relationship between the GPx‐1 rs1800668 allele and personality traits. The NS2 scores in women were significantly different in each genotype (*X* ^2^ = 8.232, *p* = .016), and the CT genotype had the highest NS2 score, indicating they may have the stranger antioxidant activity ability and be better protected against oxidative stress (Tondreau et al., 2012). Compared with males, females also had higher erythrocyte GPx activity confirms earlier studies (Donadio et al., 2016), probably because of high estrogen levels and using of hormonal contraceptive (Massafra et al., 2002; Wang et al., 2020). We found that for rs1800668, TPQ scores were higher in the statistically significant items both for overall and sex‐stratified CT genotypes, which may be associated with increased GPx enzymatic activity (Vecchio & Curro, 2017).

It should be noted that there were a few limitations to this study. First, the sample size was relatively small, but we can expand it to further explore relationships between rsl800668 and personality traits. Second, in the future, we will draw reliable conclusions by combining molecular imaging or other imaging examinations with personality evaluations. Moreover, all subjects in this study were medical staff and students in hospital and lacking the analysis of the variation in extracellular GPx1 levels. Further study is needed to verify and refine our conclusions.

## 5 CONCLUSION

This is the first study to observe that the GPx‐1 polymorphisms rs1050450 and rs1800668 were associated with the personality trait NS in a healthy Chinese‐Han population. The results showed the GPx‐1 variant rs1050450 was significantly associated with the disorderliness trait. We also found a correlation between rs1800668 and the impulsiveness trait. Our results are consistent with the suggested mechanisms of forming personality traits and will improve our understanding of the neurobiological mechanisms that are involved in shaping personality traits.

## CONFLICT OF INTEREST

None.

## AUTHOR CONTRIBUTIONS

Gang ZHU designed and corrected this paper; Xiaojun Shao collected data and wrote this paper; Dongxue Sun, Bihui Zhang, and Lingfei Cheng collected data; Ci Yan analyzed data.

## COMPLIANCE WITH ETHICAL STANDARDS

Approval was obtained from the ethics committee of China Medical University. The procedures used in this study adhere to the tenets of the Declaration of Helsinki.

## CONSENT TO PARTICIPATE

Informed consent was obtained from all individual participants included in the study.

## CONSENT TO PUBLISH

The participant has consented to the submission of the case report to the journal.

### Peer Review

The peer review history for this article is available at https://publons.com/publon/10.1002/brb3.1897.

## Data Availability

The data that support the findings of this study are available from the corresponding author upon reasonable request.
